# CAPG serves as a prognostic biomarker and promotes proliferation and migration in pancreatic ductal adenocarcinoma

**DOI:** 10.1371/journal.pone.0346011

**Published:** 2026-03-31

**Authors:** Zhongyu Qin, Kaixia Li, Xuanjie Li, Haorui Wang, Yiqiang Zhang

**Affiliations:** Department of Basic Medicine, Changzhi Medical College, Changzhi, Shanxi, China; Weill Cornell Medicine, UNITED STATES OF AMERICA

## Abstract

The actin-binding protein CAPG (Capping Actin Protein, Gelsolin Like) is implicated in oncogenesis, but its role in pancreatic ductal adenocarcinoma (PDAC) remains unclear. This study combined bioinformatic analysis of TCGA/GEO datasets, immunohistochemistry on clinical samples, and functional in vitro assays to define CAPG’s significance in PDAC. We found CAPG significantly overexpressed in PDAC tissues (n = 179 tumor vs. 171 normal, p < 0.05), with levels correlating with advanced tumor stage (T3 vs. T1) and predicting poorer overall (p = 0.0085) and disease-free (p = 0.015) survival. In vitro, siRNA-mediated CAPG knockdown in PANC-1 and AsPC-1 cells markedly inhibited proliferation (CCK-8 assay) and migration (wound healing assay), and significantly sensitized cells to gemcitabine-induced apoptosis. Mechanistically, CAPG knockdown was associated with reduced ERK1/2 phosphorylation and Cyclin D1 expression, and ERK1/2 inhibition phenocopied the anti-proliferative and chemosensitizing effects. Our results establish CAPG as a negative prognostic biomarker in PDAC, demonstrate its critical role in driving proliferation and migration—potentially via modulating ERK pathway activity—and highlight its promise as a therapeutic target whose inhibition can enhance chemotherapy efficacy.

## Introduction

Pancreatic ductal adenocarcinoma (PDAC) is a major oncological challenge [1,2]. It is often diagnosed at an advanced stage, leading to a poor prognosis and limited surgical interventions [3]. The pancreas's deep anatomical location, coupled with the absence of early detection tools, complicates diagnosis [4]. Upon symptom onset, clinicians typically resort to standard imaging tests like ultrasound, CT, MRI, and endoscopic ultrasound (EUS) [5]. However, biomarker testing, such as CA19−9, has limited utility due to sensitivity and specificity issues [6]. Recent treatment advancements have centered on immunotherapy and more effective chemotherapy regimens, aiming to improve systemic control and patient response rates [1,7]. Research is now focused on developing early-detection methods, including innovative blood tests, to identify PDAC at a more treatable stage. Looking forward, the emphasis is on early detection, refining treatment approaches, and tailoring personalized medicine strategies to combat this formidable disease.

Actin-binding proteins (ABPs) are pivotal regulators of the actin cytoskeleton, essential for cellular functions such as movement, division, and apoptosis [8,9]. These proteins play a significant role in the stages of carcinogenesis. For instance, Twinfilin Actin Binding Protein 1 (TWF1) has been identified as a potential oncogenic driver in lung, pancreatic, and breast cancers [10]. The reorganization of the actin cytoskeleton by ABPs facilitates cancer cell proliferation, migration, invasion, and metastasis, positioning them as prime targets in cancer research [11]. CAPG, belonging to the gelsolin superfamily, is implicated in the progression of various cancers, including gastric, breast, lung, liver, and prostate cancers [12]. Its involvement in cell migration and proliferation, particularly through the Wnt/β-catenin signaling pathway—a key player in cell fate determination and oncogenesis—highlights its role in tumor progression [13]. The consistent upregulation of CAPG across multiple cancer types underscores its potential as a therapeutic target, offering new avenues for treatment strategies.

Recent advancements in CAPG research have notably identified its utility as a biomarker for early gastric cancer (EGC), enhancing our understanding of cancer cell-regulating signaling pathways [13]. The discovery of CAPG-171aa, a novel polypeptide encoded by circCAPG, has been particularly significant in triple-negative breast cancer (TNBC), highlighting CAPG's potential in developing both prognostic biomarkers and therapeutic targets [14]. CAPG's interactions with proteins such as LCRMP-1, known for enhancing invasion, elucidate a complex network of pathways that influence cancer cell invasion and metastasis, thus opening new treatment avenues [15,16]. However, the exploration of CAPG's role in pancreatic cancer (PDAC) diagnosis and prognosis remains an active area of investigation.

In our study, we initially assessed CAPG expression across various cancers utilizing TCGA and GEO databases. Subsequently, we focused on CAPG expression in PDAC patients and delved into its function in PDAC cell lines following CAPG knockdown, examining the underlying mechanisms. Our findings aim to provide comprehensive insights into the prognostic and therapeutic implications of CAPG for PDAC patients, potentially guiding future research and clinical applications.

## Materials and methods

### Data collection and analysis

Gene expression profiles (RNA-Seq) and matched clinical data for pancreatic adenocarcinoma (PAAD) were downloaded from The Cancer Genome Atlas (TCGA) data portal. The cohort consisted of 179 primary tumor samples with available data, collected between December 2021 and July 2023. The clinical variables retrieved and utilized in subsequent analyses included: age at diagnosis, gender, vital status, overall survival (OS) time, tumor stage (AJCC), and tumor grade (S1 Table). Cases with incomplete survival or staging information were excluded from the corresponding analyses. Additionally, gene expression data for paired PDAC tumor and adjacent normal tissues were retrieved from the Gene Expression Omnibus (GEO, https://www.ncbi.nlm.nih.gov/geo/) under accession number GSE183795. This dataset comprises 139 pancreatic tumor tissues, 102 adjacent non-tumor tissues, and 3 normal pancreas tissues collected from September 2021 to December 2022.

To evaluate CAPG expression across various cancer types and corresponding normal tissues, GEPIA2 (Gene Expression Profiling Interactive Analysis, http://gepia2.cancer-pku.cn/#index) and GENT2 platforms (http://gent2.appex.kr/gent2/) were utilized to generate a pan-cancer CAPG expression profile. For PDAC -specific analysis, GEPIA2 was further employed. Tumor data were selected from TCGA-PAAD, and normal tissue data were sourced from matched TCGA and GTEx (https://www.gtexportal.org/home/) datasets. A violin plot was generated to visualize CAPG expression differences between PDAC tumor and normal tissues.

For the GEO dataset GSE183795, GraphPad Prism software was used to perform differential expression analysis of CAPG between normal and cancerous tissues. The results were visualized using a dot plot to illustrate the distribution and statistical significance of CAPG expression levels.

### Differential gene analysis and volcano plot construction

The GEO2R analysis tool was employed to perform differential gene expression analysis on the GSE183795 dataset. Adjacent normal tissues were designated as the control group, and tumor tissues as the experimental group. Analysis was conducted under the default settings of the website, resulting in a volcano plot that illustrates the differential gene expression outcomes. Differentially expressed genes were identified based on the criteria of log_2_ fold change > 2 and p < 0.1, which were considered statistically significant.

### Functional Enrichment Analysis

Pearson correlation analysis was performed to identify the top 500 genes most strongly correlated with CAPG expression in both the TCGA and GSE183795 datasets. The resulting gene lists were submitted to the Database for Annotation, Visualization, and Integrated Discovery (DAVID) for functional enrichment analysis. Official gene symbols were used as identifiers, and Homo sapiens was specified as the species.

Enrichment analysis was conducted to identify significant Gene Ontology (GO) terms and Kyoto Encyclopedia of Genes and Genomes (KEGG) pathways associated with CAPG-correlated genes (S2 Table). Results were visualized as bar plots, ranked in descending order based on p-values.

### Gene Set Variation Analysis (GSVA)

Gene sets related to key cellular processes—including cell movement, cell adhesion, and apoptosis—were retrieved from the AmiGO 2 Gene Ontology database (https://amigo.geneontology.org/amigo). The GSVA package (2.4.4) in R (4.5.2) was applied to expression matrices from both the TCGA and GSE183795 datasets to compute enrichment scores for each sample. The calculated GSVA scores were visualized using heatmaps generated via TBtools. All data were row-normalized, and samples were sorted in ascending order of CAPG expression to facilitate interpretation of enrichment trends.

### Cell culture

The human pancreatic cancer cell lines PANC-1 and AsPC-1 were obtained from the Chinese Academy of Sciences. Cells were cultured in Dulbecco’s Modified Eagle Medium (DMEM) (Hyclone, China) supplemented with 10% fetal bovine serum (FBS) (Gibco, USA), 100 IU/mL penicillin, and 100 μg/mL streptomycin (Hyclone, China). Cultures were maintained in a humidified incubator at 37°C with 5% CO₂.

### CAPG knockdown

PANC-1 and AsPC-1 cells were transfected with either CAPG-siRNA-1 (sense: CUGCCAUCAAGAAACUCUATT; antisense: UAGAGUUUCUUGAUGGCAGTT), CAPG-siRNA-2 (sense: GCAGCUCUGUAUAAGGUCUTT; antisense: AGACCUUAUACAGAGCUGCTT), or a negative control siRNA (sense: UUCUCCGAACGUGUCACGUTT; antisense: ACGUGACACGUUCGGAGAATT) when the cell confluence reached approximately 60%. Transfections were performed according to the manufacturer's instructions. All siRNA sequences were synthesized by Sangon Biotech (Shanghai, China). Knockdown efficiency was verified by Western blot analysis.

### RT-PCR

Quantitative reverse transcription PCR (RT-PCR) was performed to evaluate CAPG mRNA expression following knockdown. Total RNA was extracted using RNAiso Plus (Takara, Japan), and reverse transcription was carried out using the PrimeScript RT Master Mix (RR036A, Takara). The following primers were used: for CAPG, the forward primer was CTGGGCATTTGCCTTGTCAGCT and the reverse primer was CAGGTGGAGATTGTCACTGATGG; for GAPDH (internal control), the forward primer was GTCTCCTCTGACTTCAACAGCG and the reverse primer was ACCACCCTGTTGCTGTAGCCAA.

### Western blot

Protein expression was assessed by Western blotting using standard protocols. Briefly, cells were lysed in RIPA buffer (Solarbio, China) supplemented with 1% protease inhibitor cocktail (Sigma, Germany) and phenylmethylsulfonyl fluoride (PMSF). After incubation for 30 minutes on ice, protein concentrations were determined using a BCA protein assay kit.

Equal amounts (20 μg) of total protein were separated by SDS-PAGE and transferred onto nitrocellulose membranes (Millipore, USA). Membranes were blocked with 5% non-fat milk and incubated overnight at 4°C with primary antibodies:CAPG (AF0013, Affinity Biosciences, China, 1:1000); p-ERK1/2 (4377s, CST, USA, 1:1000); ERK1/2 (5013s, CST, USA, 1:1000); Cyclin D1 (A19038, Abclonal, USA, 1:2000); β-actin (4970, CST, USA, 1:1000). After washing, membranes were incubated with HRP-conjugated secondary antibodies (S0001, Affinity Biosciences, China, 1:3000) at room temperature for 1 hour. Protein bands were visualized using an enhanced chemiluminescence (ECL) kit (Beyotime, China) and quantified using ImageJ software.

### Cell proliferation assay

A total of 1,000 PANC-1 or AsPC-1 cells transfected with either si-NC or si-CAPG were seeded into 96-well plates. Where indicated, cells were treated with LM22B‑10 (ERK agonist, 0.1μM, MCE: HY-104047) at the indicated concentration; vehicle controls received matched DMSO (final DMSO ≤0.1%). After cell attachment, 10% CCK-8 solution (Solarbio, China) was added to each well at 24, 48, 72, 96 and 120 hours, followed by incubation at 37°C for 2 hours. Cell proliferation was quantified by measuring absorbance at 450 nm using a microplate reader. In addition, proliferating tumor cells were evaluated using Ki67 staining (350521; BioLegend, USA) combined with flow cytometry analysis. Briefly, cells were harvested, fixed and permeabilized in 70% ethanol at 4°C for 30 minutes. Subsequently, the cells were incubated with a fluorescein-conjugated anti-Ki67 antibody (diluted 1:50 in permeabilization buffer) for 30 minutes at 4°C in the dark. After washing, the stained cells were analyzed by flow cytometry, and the percentage of Ki67-positive cells was determined.

### Cell wound healing assay

The migratory capacity of pancreatic cancer cells following CAPG knockdown was assessed using a wound healing assay. Briefly, transfected cells were seeded into 96-well plates and cultured until they reached approximately 90% confluence. A uniform scratch was then created across the cell monolayer in both the experimental and control groups. Detached cells were removed by washing with phosphate-buffered saline (PBS), after which the medium was replaced with serum-free medium. Cell migration was monitored at 0 and 12 hours using a microscope, and migration distances were quantified with ImageJ software.

### Apoptosis assay

PANC-1 and AsPC-1 cells, including their CAPG-silenced counterparts and those pretreated with the ERK inhibitor PD98059 (20 µM, 12 h), were treated with 10 nM gemcitabine for 24 hours. After treatment, cells were digested, collected, and stained using an Annexin V/propidium iodide (PI) apoptosis detection kit (C1062S, Beyotime, China) according to the manufacturer’s protocol. Caspase-3/7 activity was measured using a fluorescent Caspase 3/7 Activity Assay Kit (E-CK-A383, China) according to the manufacturer’s protocol. The proportion of apoptotic and dead cells was subsequently determined by flow cytometry.

### Immunohistochemistry

A paraffin-embedded human pancreatic cancer (PDAC) tissue microarray (five paired tumor and adjacent normal tissues) was purchased from Shanghai Outdo Biotech Company (Shanghai, China). The use of these de-identified archived specimens was reviewed and approved by the Ethics Committee of Shanghai Outdo Biotech Company (Approval No. SHYJS-CP-1810012). Sections were deparaffinized in xylene and rehydrated through graded ethanol, followed by antigen retrieval in citrate buffer (pH 6.0). After blocking endogenous peroxidase activity (3% H_2_O_2_) and nonspecific binding (5% normal serum), sections were incubated overnight at 4°C with an anti-CAPG primary antibody (AF0013, Affinity Biosciences, China, 1:200). Visualization was performed using an HRP–DAB detection kit (Vector, SK4100), and nuclei were counterstained with hematoxylin. CAPG staining scores were calculated using ImageJ software by quantifying the percentage of positively stained cells.

### Immunofluorescence

To assess the effect of CAPG knockdown on cell proliferation, an EdU staining assay was performed. Briefly, cells were seeded in 24-well plates and cultured until they reached approximately 50% confluence. The cells were then fixed and permeabilized using Intracellular Fixation/Permeabilization Buffer (eBioscience, USA). EdU staining solution (1 μM, C0071S, Beyotime, China) was added, and the cells were incubated in the dark for 15 minutes. After three washes with PBS, EdU-positive cells were visualized under a fluorescence microscope.

### Statistical analysis

SPSS 27.0 software was used to perform univariate and multivariate Cox proportional hazards regression analyses. CAPG expression data from the TCGA database, along with various clinical data from patients, were analyzed using both univariate and multivariate models. Results were considered statistically significant at P < 0.05, P < 0.01, and P < 0.001. All cell-based experiments were repeated three times.

## Results

### CAPG is highly expressed in PDAC tumor tissues

**Fig 1:**
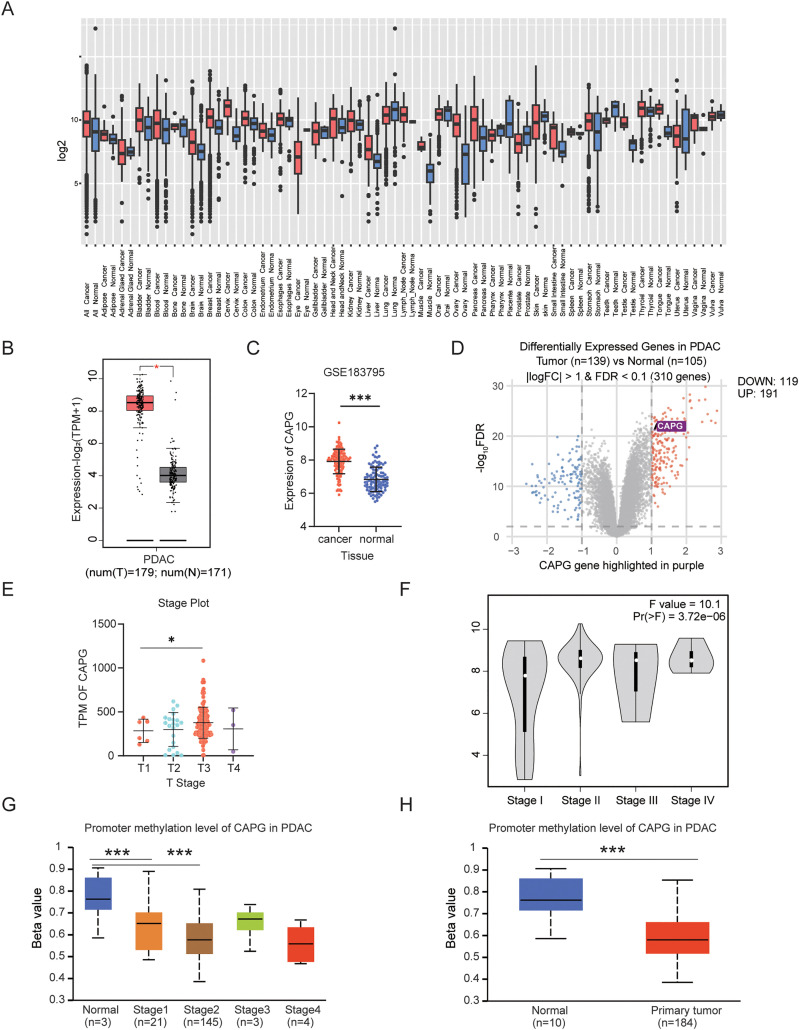
CAPG is highly expressed in clinical samples from PDAC patients. A–B: The gene expression levels of CAPG across various tissues were analyzed using the GEPIA2 and GENT2 platforms, comparing tumor tissues with adjacent non-tumor tissues. P-values below 0.05 indicate statistical significance. C: Differential expression of CAPG between PDAC tumor tissues and adjacent non-tumor tissues was assessed using data from the GEPIA2 and GSE183795 databases. The GEPIA2 expression data were obtained from the TCGA dataset. D: A differential gene expression analysis was performed using GEO2R on the GSE183795 dataset, and the results were visualized as a volcano plot. The location of CAPG is indicated on the plot. E–F: A dot plot illustrates CAPG expression under different T-stages (data sourced from TCGA; ANOVA summary: F = 1.644, P = 0.1813). The violin plot, generated via GEPIA2, displays CAPG expression across various pathological stages. G: The promoter methylation status of CAPG across different stages of pancreatic cancer was analyzed using the UALCAN platform, with p-values shown in the accompanying table. H: CAPG promoter methylation levels were compared between pancreatic cancer cells and normal pancreatic cells using UALCAN, with detailed p-values also provided in the table.

To assess the correlation between CAPG expression and pancreatic cancer (PDAC) patient outcomes, we initiated our analysis using pan-cancer data from the TCGA and GEO databases. Our findings indicate a significant overexpression of CAPG across various cancers, including brain, cervix, liver, muscle, ovary, prostate, testis, and pancreatic cancer (Fig 1A).

In a comparative analysis, CAPG was found to be significantly elevated in 179 tumor samples compared to 171 matched normal adjacent tissues (Fig 1B). This observation was further substantiated by GEO datasets, which confirmed markedly elevated CAPG expression in pancreatic cancer tumor sites (Figs 1C-1D).

Analysis of tumor stages revealed that CAPG expression levels were higher in stage T3 compared to T1, suggesting that CAPG expression increases with tumor progression (Figs 1E-1F). Given the known inverse relationship between CAPG methylation and its expression, we examined the methylation status of the CAPG promoter across different PDAC stages. Our results demonstrated a significant reduction in CAPG methylation in tumor tissues compared to normal tissues (Figs 1G-1H), implying a regulatory mechanism for CAPG expression in PDAC.

### CAPG regulates biological processes associated with cell division and adhesion

**Fig 2:**
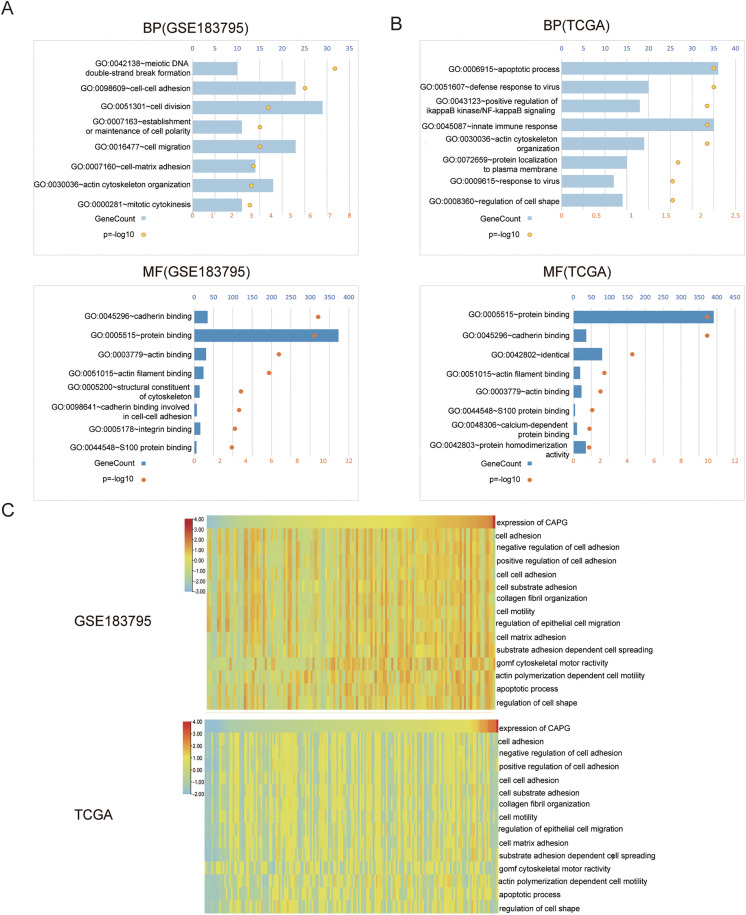
Correlation Between CAPG Expression and Functional Enrichment Scores in Cancer Cells. A–B: The biological processes (BP) and molecular functions (MF) most strongly associated with CAPG expression were identified using the GEO (A) and TCGA (B) datasets. C: A heatmap displays the correlations between CAPG expression and enrichment scores of various cellular functions in the GEO and TCGA datasets, as determined by Gene Set Variation Analysis (GSVA).

To elucidate the biological functions associated with CAPG, we performed a Pearson correlation analysis using data from the GEO and TCGA databases to identify genes closely related to CAPG. KEGG pathway analysis of these gene sets revealed that, in the GEO database, the biological processes most strongly associated with CAPG include cell division, cell adhesion, and protein binding (Fig 2A). Additionally, the analysis highlighted CAPG's significant involvement in the apoptotic process, innate immune response, and protein binding within the TCGA database (Fig 2B).

Furthermore, we employed Gene Set Variation Analysis (GSVA) across both the GEO and TCGA databases to calculate enrichment scores for samples with high CAPG expression. This analysis pinpointed that pathways related to cell adhesion are notably enriched in samples expressing high levels of CAPG (Fig 2C).

### CAPG as a prognostic marker for pancreatic cancer

**Fig 3:**
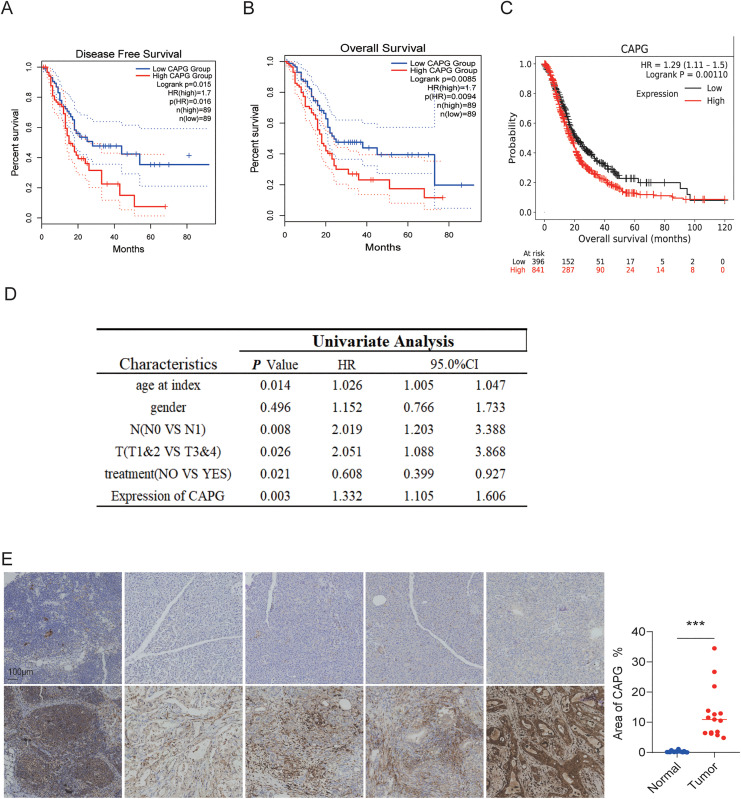
High CAPG Expression Is Associated with Shorter Overall Survival in Pancreatic Cancer (PDAC). A–C: Kaplan-Meier survival analysis was performed to assess the impact of CAPG expression on disease-free survival (A) and overall survival (B) in PDAC patients, using data from the TCGA database, as well as the GEO database **(C)**. Red: high CAPG expression; Blue: low CAPG expression. D: Univariate analysis was conducted to evaluate prognostic factors, including age, lymph node metastasis, tumor stage, and treatment modalities, using the TCGA database. E: Immunohistochemistry (IHC) staining was used to detect CAPG expression in PDAC tumors and adjacent normal tissues. Representative images are shown. Quantitative analysis summarizes data from 5 paired samples; three fields of view were selected for each sample, resulting in a total of 15 paired images analyzed. Statistical significance was calculated using a t-test (***p < 0.001).

CAPG has been identified as a potential prognostic marker for PDAC. Previous findings have demonstrated that CAPG is highly expressed in PDAC, and this expression positively correlates with the tumor stage [17]. To further explore the prognostic significance of CAPG in PDAC, we utilized the GEO dataset to analyze patient survival. Our analysis revealed that patients with high CAPG expression exhibited a shorter overall survival rate (high: n = 89, low: n = 89, p = 0.0085) and disease-free survival (high: n = 89, low: n = 89, p = 0.015) (Fig 3A-3B). These findings were corroborated by KM-plot analysis, which showed that patients with high CAPG expression had significantly lower survival times (Fig 3C). In an effort to assess the prognostic utility of CAPG in PDAC, we examined the relationship between CAPG expression and various clinical characteristics, including histologic subtypes, clinical stages, and grades of PDAC, using the Kaplan-Meier Plotter online tool. Our study found that CAPG expression is positively correlated with advanced clinicopathological features, including older age, lymph node metastasis (N1 status), and higher tumor stage (T3 and T4) (Fig 3D). Consistently, IHC staining of human PDAC samples confirmed higher CAPG expression in tumor sites compared to adjacent normal tissue (Fig 3E).

### The Role of CAPG in Promoting PC Tumor Cell Proliferation

**Fig 4:**
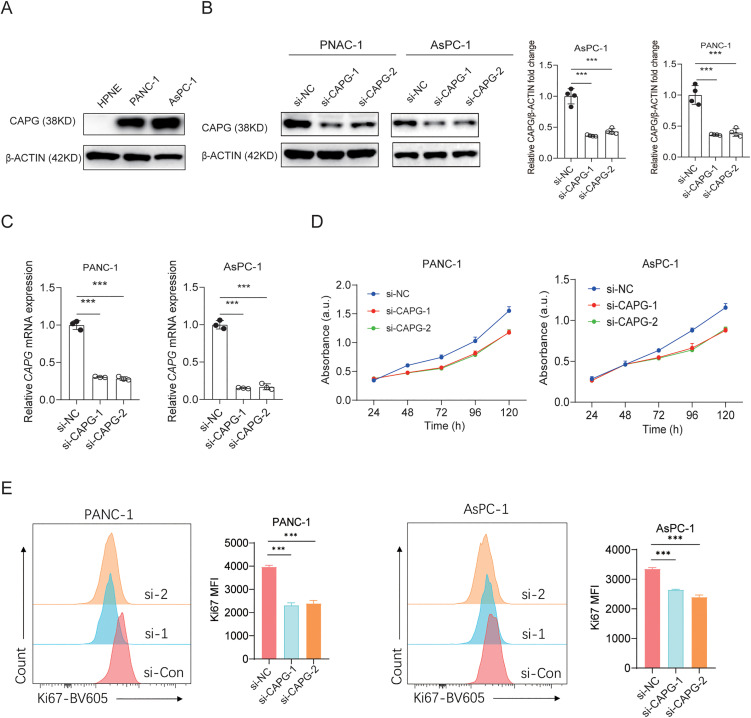
Inhibition of Pancreatic Cancer (PDAC) Cell Proliferation by CAPG Knockdown. A: Western blot analysis was performed to examine CAPG expression levels in HPNE, PANC-1, and AsPC-1 cell lines. B: Western blot analysis was conducted to evaluate CAPG protein levels, with β-actin used as a loading control. The results were quantified and presented in a bar plot. C: Quantitative RT-PCR was used to measure CAPG mRNA expression. D: Cell proliferation was assessed at various time points using the CCK-8 assay. E: Flow cytometry analysis was performed to evaluate cell proliferation via Ki67 staining. Representative images and corresponding bar plots depict the Mean Fluorescence Intensity (MFI) of Ki67 expression in each cell line. Statistical analyses were performed using one-way ANOVA for panels B, C, and E, and a t-test for panel **D.** Significance is indicated as follows: *p < 0.01; ***p < 0.001.

To investigate the role of CAPG in promoting the proliferation of pancreatic cancer (PDAC) tumor cells, we first examined CAPG expression levels in human pancreatic duct cell lines (HPNE) and PDAC tumor cell lines (PANC-1 and AsPC-1). Our results showed that CAPG was significantly upregulated in the PC tumor cell lines compared to HPNE (Fig 4A).

Subsequently, we employed siRNA to knock down CAPG in both PANC-1 and AsPC-1 cells. The successful reduction of CAPG expression was confirmed through RT-PCR and Western blot analyses (Fig 4B-4C). We then utilized the CCK-8 assay to assess cell proliferation, revealing that CAPG knockdown markedly inhibited the proliferation of both PANC-1 and AsPC-1 cells (Fig 4D).

To further investigate whether the pro-survival TrkB/TrkC-AKT/ERK signaling pathway is involved in the proliferative role of CAPG, we performed a rescue experiment using LM22B-10, a specific agonist of TrkB/TrkC receptors. Consistent with its known pro-survival function, LM22B-10 treatment exhibited a clear survival-promoting effect, as evidenced by an overall upward shift in the CCK-8 growth curves. Notably, this effect was most pronounced in the context of CAPG knockdown. In both PANC-1 and AsPC-1 cells, LM22B-10 partially but significantly rescued the proliferation deficit caused by CAPG silencing, bringing the growth curves closer to those of the control (si-NC) groups. In contrast, the impact of LM22B-10 on si-NC control cells was minimal or non-significant (S1 Fig). These consistent findings across both cell lines support the mechanistic premise that CAPG promotes proliferation/survival, at least in part, through pathways that can be activated by TrkB/TrkC signaling.

This inhibitory effect on cell proliferation was further validated by Ki67 staining, which showed a significant reduction in Ki67 levels following CAPG knockdown (Fig 4E).

### CAPG promotes migration and its knockdown sensitizes pancreatic cancer cells to gemcitabine

**Fig 5:**
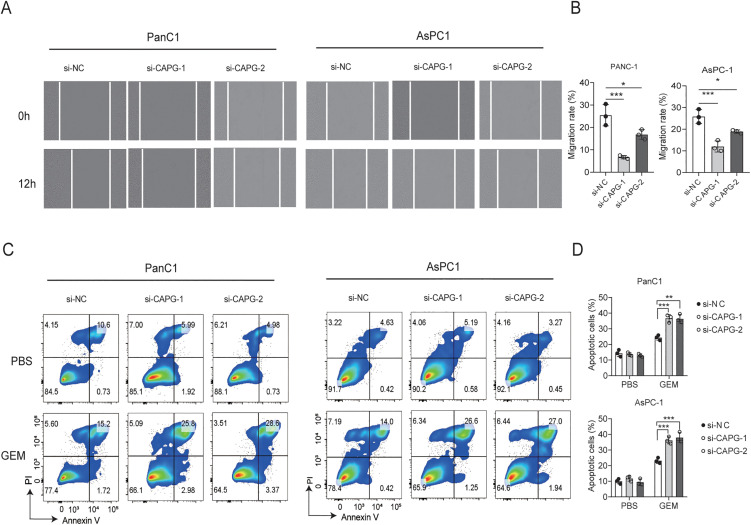
CAPG Promotes Migration and Its Knockdown Sensitizes Pancreatic Cancer Cells to Gemcitabine-induced apoptosis. A–B: A wound healing assay was performed to assess the migratory capacity of PANC-1 and AsPC-1 cells following CAPG knockdown. Images were captured at 0 and 12 hours to visualize wound closure, and the migration rates were quantified and shown in bar plots. C–D: After 24-hour treatment with gemcitabine (GEM, 10 nM), apoptotic cells in PANC-1 and AsPC-1 lines were detected using an Annexin V/PI staining kit. Representative flow cytometry plots and corresponding bar graphs display the percentage of apoptotic cells in each group. Data are presented as mean ± SD. Statistical analyses were performed using one-way ANOVA, with significance indicated as *p < 0.01, ***p < 0.001.

In our investigation into the impact of CAPG on PDAC cell migration, we employed a wound healing assay to measure cell coverage. Our cell wound healing assay results indicated that the knockdown of CAPG in both PANC-1 and AsPC-1 cells significantly inhibited cell migration (Fig 5A-5B). Furthermore, we examined apoptosis in PANC-1 and AsPC-1 cells following treatment with the chemotherapy drug gemcitabine. We found that CAPG knockdown alone did not affect the apoptosis rate; however, it significantly increased the cells’ sensitivity to gemcitabine (10 nM, 24 h), resulting in a higher apoptosis rate in these PDAC cell lines (Fig 5C-5D). Similarly, a Caspase-3/7 assay confirmed that CAPG knockdown significantly enhanced sensitivity to gemcitabine (S2 Fig)

### CAPG knockdown is associated with reduced ERK1/2 phosphorylation and Cyclin D1 expression

**Fig 6:**
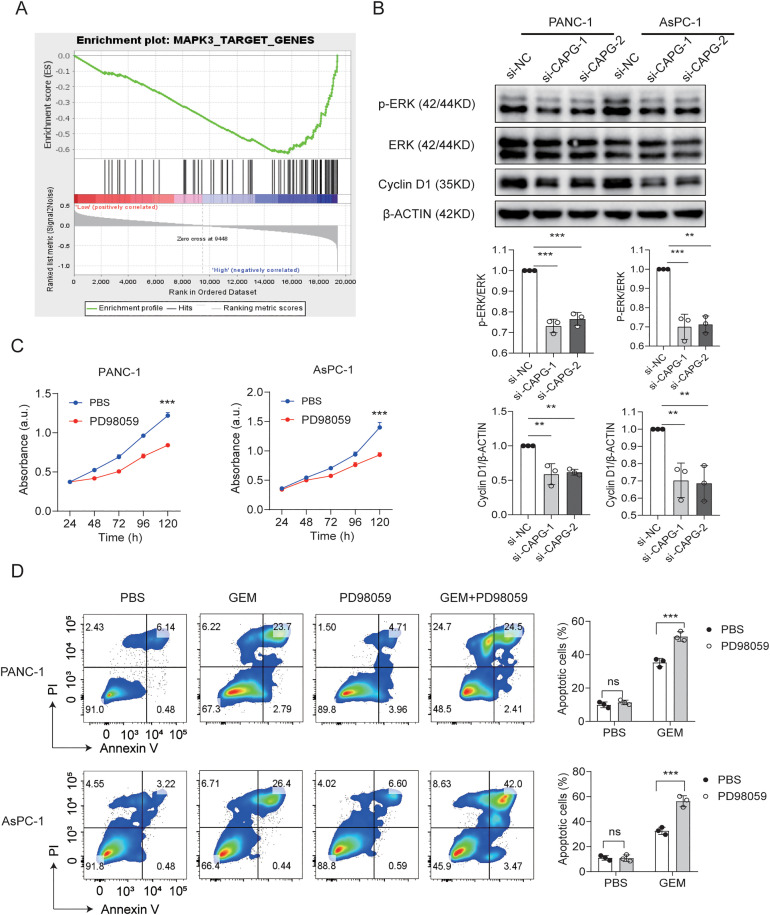
CAPG Knockdown Reduces ERK1/2 Phosphorylation and Cyclin D1 Expression. A: GSEA enrichment plots show that MAPK3-related gene sets are significantly enriched in the CAPG high- and low-expression groups within the GEO dataset. B: Western blot analysis was used to assess the expression of phosphorylated ERK (p-ERK) and Cyclin D in PANC-1 and AsPC-1 cells following CAPG knockout, with β-actin as a loading control. Representative blot images and quantification data are provided. C: Cell proliferation was measured at various time points using the CCK-8 assay. D: The ERK1/2 inhibitor PD98059 (20 μM) was administered to PANC-1 and AsPC-1 cells, either alone or in combination with gemcitabine (GEM, 10 nM), for 24 hours. Apoptotic cells were analyzed by flow cytometry. Representative images and bar plots illustrate the percentage of apoptotic cells. Statistical analysis was performed using two-way ANOVA. Significance levels are indicated as *p < 0.01, ***p < 0.001.

Our data have demonstrated that CAPG plays a crucial role in promoting the proliferation and migration of PDAC cells. To uncover the underlying mechanisms, we analyzed gene expression differences between samples with high and low CAPG expression. GSEA analysis indicated a significant enrichment of MAPK3-related genes, which are known to contribute to cell cycle regulation (Fig 6A). Western blot analysis revealed that knocking down CAPG significantly reduced the phosphorylation of ERK and the levels of the downstream cell cycle protein, Cyclin D1 (Fig 6B). Furthermore, the application of an ERK1/2 inhibitor PD98059 markedly suppressed the proliferation of PANC-1 and AsPC-1 cells (Fig 6C), whereas the ERK agonist LM22B-10 reversed the reduction in cell proliferation induced by CAPG knockdown (S1 Fig). Moreover, treatment of PD98059 significantly increased the sensitivity to gemcitabine (Fig 6D). Based on these findings, we propose that CAPG may enhance PC cell proliferation via the activation of the p-ERK/Cyclin pathway.

## Discussion

Pancreatic ductal adenocarcinoma (PDAC) is a highly aggressive malignancy characterized by poor prognosis and high global mortality rates [5]. Uncontrolled proliferation is a hallmark of PDAC, posing a significant threat to human health [18]. Previous studies have suggested that CAPG, an actin-binding protein, performs key biological functions in multiple tissues, including gastric, lung, breast, liver, and prostate cancers [16]. However, its role in the development of PDAC has not been fully investigated. In this study, we have made significant strides in elucidating the role of CAPG in PDAC. By combining GSEA, GEO dataset analysis, and IHC staining, we observed significantly elevated CAPG expression in PDAC samples compared to adjacent normal tissues. This overexpression, consistent with its proposed oncogenic function in other cancers, was positively correlated with advanced tumor stages. Our findings are consistent with a model in which CAPG contributes to PDAC aggressiveness, potentially through mechanisms involving the p-ERK/cyclin D axis, thereby underscoring CAPG's potential as a prognostic biomarker and therapeutic target for PDAC.

The tumor-promoting role of CAPG has been reported in various malignancies. Our analysis of TCGA and GEO datasets confirmed that CAPG is highly expressed in various cancers, including brain, cervix, liver, muscle, ovary, pancreas, prostate, and testis. Moreover, CAPG expression in PDAC is associated with tumor stage, indicating its contribution to tumor development. Survival analysis indicated that patients with high CAPG expression exhibited shorter overall survival, supporting its potential as a prognostic biomarker and therapeutic target. This is consistent with previous reports in colon cancer, breast cancer, and diffuse large B-cell lymphoma [19–23]. Further analysis should increase patient numbers and deeply analyze clinical features, including histology, gender, lymph node metastasis, smoking history, and treatment history, to understand the relevance of CAPG expression.

CAPG, a member of the gelsolin superfamily, regulates actin filament growth in a Ca^2+^-dependent manner and is associated with tumor development [24,25]. Overexpression of CAPG has been shown to promote the progression of gastric cancer via the Wnt/β-catenin signaling pathway [13]. In this study, functional assays provided evidence that CAPG contributes to PDAC cell aggressiveness, enhancing both proliferative and migratory capacities in PANC-1 and AsPC-1 cell lines. This is further supported by the observation that CAPG knockdown significantly reduces PDAC cell proliferation, highlighting its potential as a therapeutic target. Moreover, our results also suggested that CAPG promotes tumor cell migration. These findings suggest that CAPG plays a crucial role in tumor cell proliferation and migration [26]. Additionally, we found that CAPG knockdown cells are more sensitive to gemcitabine-induced apoptosis, indicating that targeting CAPG expression may enhance chemotherapy efficacy. However, this requires further investigation.

The mechanistic insights gained from this study are particularly noteworthy. Multiple studies have indicated that excessive or inappropriate activation of the ERK pathway is implicated in the initiation and progression of human cancers [27–29]. Our GSEA identified ERK1/2 phosphorylation as a critical mediator of CAPG-driven proliferation, consistent with the established role of the ERK1/2 signaling pathway in cell proliferation and survival [30]. Preclinical studies suggest that ERK inhibitors effectively inhibit BRAF/RAS-mutated tumor growth and overcome resistance to BRAF or MEK inhibitors [31,32]. This aligns with the hypothesis that CAPG's pro-tumorigenic effects may be, in part, ERK-dependent. Notably, treatment with the ERK1/2 inhibitor PD98059 significantly enhanced gemcitabine-induced apoptosis, highlighting the need for future studies to explore the combined effect of CAPG inhibition and ERK1/2 blockade in vivo. Additionally, the translational aspect of these findings needs to be addressed through clinical trials to assess the efficacy of CAPG-targeted therapies in patients.

## Conclusions

In conclusion, this study highlights a significant role for CAPG in PDAC progression. Our data strongly implicate CAPG in promoting tumor cell migration and are consistent with a role in regulating cell proliferation. The association between CAPG manipulation and ERK1/2 pathway activity suggests that CAPG may function upstream of ERK1/2 signaling, generating a testable hypothesis for future mechanistic studies. These insights inform the development of novel therapeutic strategies for PDAC. Future research should directly test the ERK-dependency through rescue experiments, explore the long-term effects and potential compensatory mechanisms following CAPG inhibition, and investigate the interplay of CAPG with other signaling pathways in PDAC biology.

### Limitations and future perspectives

Several limitations in this study warrant mention. First, our functional assays relied primarily on in vitro models, which cannot fully recapitulate the complex PDAC tumor microenvironment. Second, our clinical validation via IHC involved a limited sample size, necessitating larger prospective cohorts to establish CAPG as a robust biomarker. Third, while we implicated the ERK/Cyclin D1 axis, the precise molecular interactions initiating this signaling cascade remain to be fully elucidated. Specifically, although our rescue experiments using the TrkB/TrkC agonist LM22B-10 suggest a potential dependency of CAPG-mediated phenotypes on the ERK1/2 signaling pathway, these findings must be interpreted with caution. As neurotrophin receptor agonists can activate pleiotropic downstream cascades (such as the PI3K/AKT pathways), pharmacological rescue of ERK signaling does not perfectly recapitulate the physiological function of intact CAPG expression. Therefore, ectopic expression of CAPG in deficient models remains the gold standard to definitively validate its pleiotropic oncogenic roles.

To address these mechanistic gaps, future investigations employing direct CAPG overexpression and highly specific genetic epistatic models are required to thoroughly disentangle the precise downstream effectors of CAPG in PC. Beyond mechanistic validations, future research should prioritize in vivo validation using patient-derived xenografts or transgenic models to confirm CAPG’s therapeutic potential in a physiological context. Additionally, given our bioinformatics findings, investigating CAPG’s influence on the immune landscape is essential. Furthermore, exploring the synergistic potential of CAPG inhibition combined with standard chemotherapies, such as gemcitabine, represents a vital avenue for translational research. Finally, detailed structural analysis of CAPG-mediated signaling could facilitate the design of specific small-molecule inhibitors for clinical application.

## Supporting information

S1 Fig CCK-8 proliferation curves of pancreatic cancer cells under the indicated treatments. CCK‑8 proliferation of PANC‑1 and AsPC‑1 cells transfected with si‑NC or si‑CAPG‑1, with or without LM22B‑10 (a TrkB/TrkC agonist that activates AKT/ERK in vitro and in vivo). Absorbance reflects proliferation over time. si‑CAPG‑1 suppresses growth; LM22B‑10 partially rescues. Mean ± SD; *** P < 0.001.
(TIF)

S2 Fig Caspase-3/7 activity assay of apoptosis levels in pancreatic cancer cells. Caspase-3/7 activity in PANC1 (left) and AsPC1 (right) cells transfected with siCon, si-CAPG1, or si-CAPG2 and treated with PBS or gemcitabine (GEM). Fluorescence-based Caspase-3/7 activity was normalized to OD490/525. Mean ± SD; *** P < 0.001.
(TIF)

S1 Table Clinicopathological Characteristics of the TCGA-PAAD Cohort. (PDF)

S2 Table All GO identifiers, and associated data. (DOCX)

S1 Raw Images Original images for blots. (PDF)
