# Supplementary figures and images for: CAPG serves as a prognostic biomarker and promotes proliferation and migration in pancreatic ductal adenocarcinoma
Source: PLoS One. 2026 Mar 31;21(3):e0346011. doi: 10.1371/journal.pone.0346011 (PMC13037992; doi:10.1371/journal.pone.0346011)

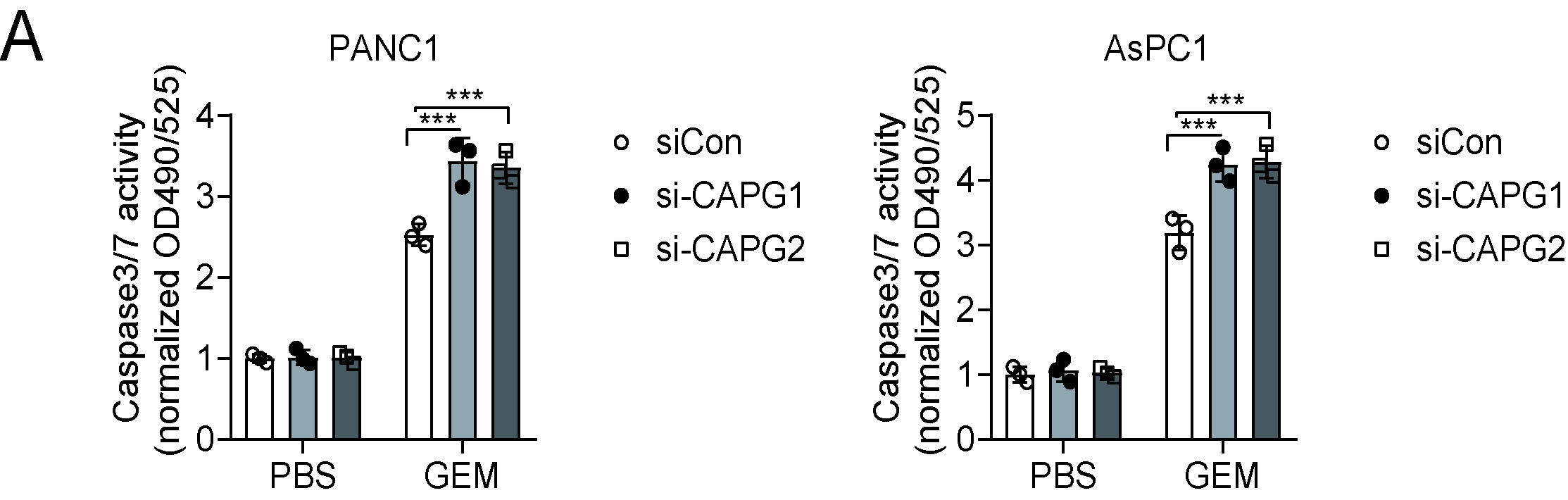

Supplement: S1 Fig — CCK‑8 proliferation of PANC‑1 and AsPC‑1 cells transfected with si‑NC or si‑CAPG‑1, with or without LM22B‑10 (a TrkB/TrkC agonist that activates AKT/ERK in vitro and in vivo). Absorbance reflects proliferation over time. si‑CAPG‑1 suppresses growth; LM22B‑10 partially rescues. Mean ± SD; *** P < 0.001. (TIF) [file pone.0346011.s001.tif]
