# Supplementary material for: CAPG serves as a prognostic biomarker and promotes proliferation and migration in pancreatic ductal adenocarcinoma
Source: PLoS One. 2026 Mar 31;21(3):e0346011. doi: 10.1371/journal.pone.0346011 (PMC13037992; doi:10.1371/journal.pone.0346011)
Supplement: S1 Table — (PDF) [file pone.0346011.s003.pdf]

A

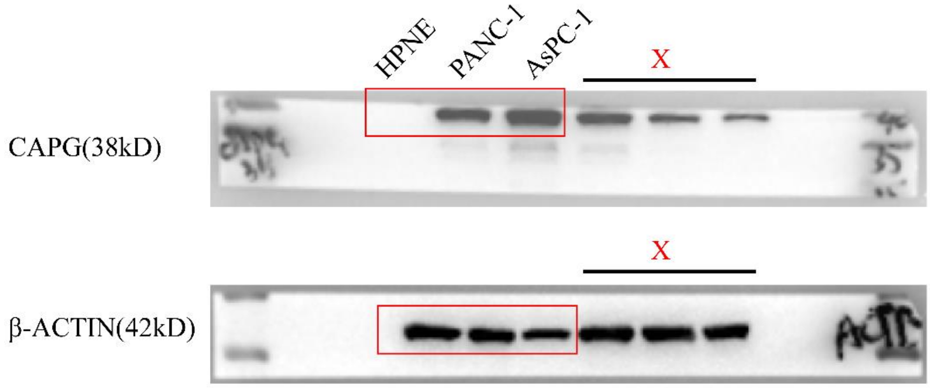

B

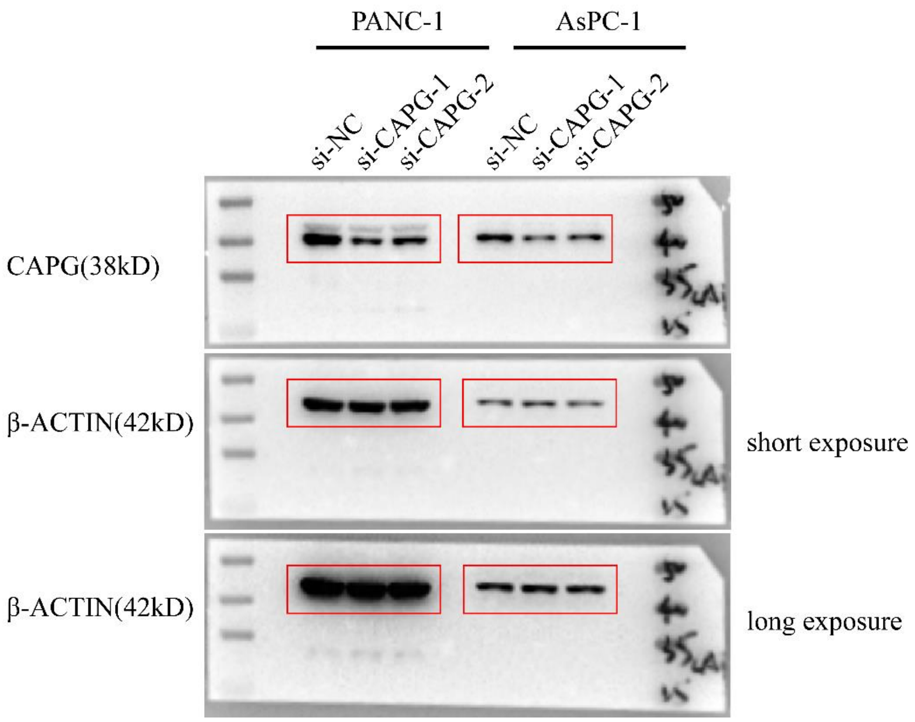

Fig 4 Original blot image. A corresponds to Fig 4A in the text, and B corresponds to Fig 4B in the text. The area enclosed by the red box is the section presented in the final figure of the paper. The loading order and sample types are consistent with those described in the text and are labeled above the image. The red "X" indicates regions not displayed in the final figure.

B

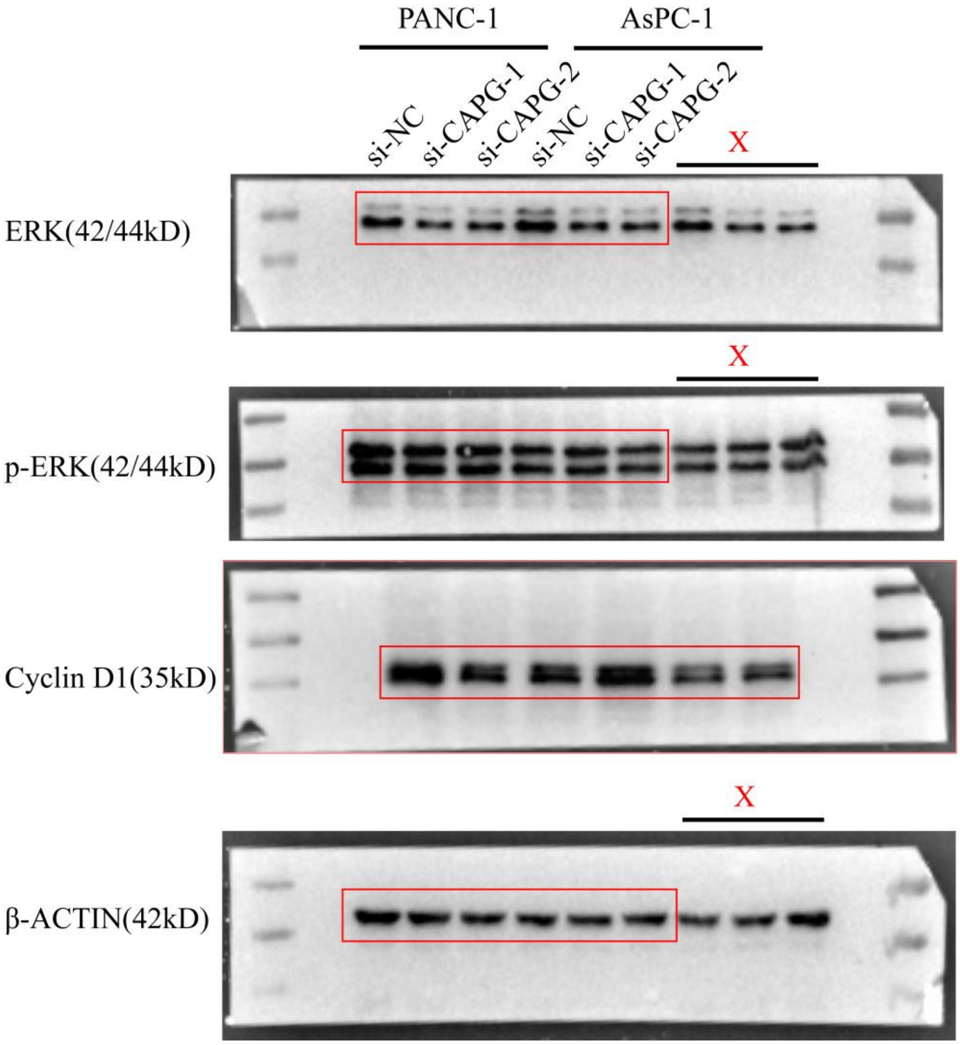

Fig 6 Original blot image. B corresponds to Fig 6B in the text. The area enclosed by the red box is the section presented in the final figure of the paper. The loading order and sample types are consistent with those described in the text and are labeled above the image. The red "X" indicates regions not displayed in the final figure.
