# Supplementary material for: CAPG serves as a prognostic biomarker and promotes proliferation and migration in pancreatic ductal adenocarcinoma
Source: PLoS One. 2026 Mar 31;21(3):e0346011. doi: 10.1371/journal.pone.0346011 (PMC13037992; doi:10.1371/journal.pone.0346011)
Supplement: S2 Table — (DOCX) [file pone.0346011.s004.docx]

**S2 Table. All GO identifiers, and associated data.**

| GO ID | Pathway Name | GSE183795 | TCGA |
| --- | --- | --- | --- |
| GO:0000281 | mitotic cytokinesis | ✔️ |  |
| GO:0003779 | actin binding | ✔️ | ✔️ |
| GO:0005178 | integrin binding | ✔️ |  |
| GO:0005200 | structural constituent of cytoskeleton | ✔️ |  |
| GO:0005515 | protein binding | ✔️ | ✔️ |
| GO:0006915 | apoptotic process |  | ✔️ |
| GO:0007160 | cell-matrix adhesion | ✔️ |  |
| GO:0007163 | establishment or maintenance of cell polarity | ✔️ |  |
| GO:0008360 | regulation of cell shape |  | ✔️ |
| GO:0009615 | response to virus |  | ✔️ |
| GO:0016477 | cell migration | ✔️ |  |
| GO:0030036 | actin cytoskeleton organization | ✔️ | ✔️ |
| GO:0042138 | meiotic DNA double-strand break formation | ✔️ |  |
| GO:0042802 | identical protein binding |  | ✔️ |
| GO:0042803 | protein homodimerization activity |  | ✔️ |
| GO:0043123 | positive regulation of I-kappaB kinase/NF-kappaB signaling |  | ✔️ |
| GO:0044548 | S100 protein binding | ✔️ | ✔️ |
| GO:0045087 | innate immune response |  | ✔️ |
| GO:0045296 | cadherin binding | ✔️ | ✔️ |
| GO:0051015 | actin filament binding | ✔️ | ✔️ |
| GO:0051301 | cell division | ✔️ |  |
| GO:0051607 | defense response to virus |  | ✔️ |
| GO:0072659 | protein localization to plasma membrane |  | ✔️ |
| GO:0098609 | cell-cell adhesion | ✔️ |  |
| GO:0098641 | cadherin binding involved in cell-cell adhesion | ✔️ |  |
| GO:0048306 | calcium-dependent protein binding |  | ✔️ |
